# Case Report of acute myeloid leukemia with “WT1, ATRX, CEBPA, CSMD1, IKZF1, and LRP1B mutation and translocation between chromosome 1 and 19” developing from Philadelphia-negative chronic myeloid leukemia after TKI therapy

**DOI:** 10.1097/MD.0000000000018888

**Published:** 2020-01-17

**Authors:** Huan Zhu, Bin Yang, Jia Liu, Wei Wu, Yun Ling

**Affiliations:** Department of Hematology, The Third Affiliated Hospital of Soochow University, The First People's Hospital of Changzhou, Changzhou, Jiangsu 213200, China.

**Keywords:** acute myeloid leukaemia, chronic myeloid leukaemia, Philadelphia chromosome-negative, tyrosine kinase inhibitor

## Abstract

**Rationale:**

The success of tyrosine kinase inhibitor (TKI) therapy has greatly prolonged the survival time of patients with chronic myeloid leukemia (CML), harboring the characteristic Philadelphia (Ph) chromosome. However, a fraction of patients, achieving complete cytogenetic response after TKI therapy, develop a myelodysplastic syndrome (MDS) or acute myeloid leukemia (AML) with additional clonal chromosomal abnormalities in Philadelphia-negative cells (CCA/Ph–).

**Patient concerns:**

A 56-year-old woman with AML, developing from Philadelphia-negative CML after TKI therapy. She showed 6 kinds of somatic variants—CEBPA, ATRX, WT1, CSMD1, IKZF1, and LRP1B mutation after diagnosed as AML.

**Diagnosis:**

The patient was diagnosed with chronic phase CML that developed to AML after achieving durable complete cytogenetic response (CCR) and major molecular response (MMR).

**Interventions:**

The patient was treated with TKI therapy at the period of CML. When diagnosed with AML, she received induction chemotherapy regimens, consolidation therapy, and allogeneic hematopoietic stem cell transplantation subsequently.

**Outcomes:**

The patient has been CCR and MMR for nearly 4 years, and has achieved complete remission after intervention related to AML. She is now preparing for allogeneic hematopoietic stem cell transplantation.

**Lessons:**

These rare occurrences highlight the importance of exploring the relevant pathogenesis of AML developing from CML after TKI therapy. In addition to monitoring molecular changes in the course of CML, cytogenetic analysis, or next-generation sequencing of CML patients should be performed.

## 1 Introduction

Chronic myeloid leukemia (CML), harboring the characteristic Philadelphia (Ph) chromosome, a translocation between chromosome 9 and 22, is associated with a significantly improved overall survival rate after tyrosine kinase inhibitor (TKI) therapy. TKIs, which originally inhibited the activity of BCR-ABL1 fusion gene product, have been performing an extremely important role in CML patients.^[1]^ In addition to the characteristic chromosomal aberration, studies related to additional clonal chromosomal abnormalities in Philadelphia-negative cells (CCA/Ph–) of CML after TKI therapy have been reported in a small subset of patients, and the ratio related to imatinib is 2% to 17%.^[2,3]^ Some of the CCA/Ph–in CML are transient, whereas others persist,^[4]^ and the influence of CCA/Ph−on the clinical course of CML is controversial. As is shown in some reports, the overall prognosis of CCA/Ph– CML is good and depends on the response to imatinib therapy.^[5]^ Rare cases of CML treated by TKIs, including imatinib, dasatinib, and nilotinib, progressing to myelodysplastic syndrome (MDS)/acute myeloid leukemia (AML) from CCA/Ph–have been reported,^[6–8]^ even though they are in complete cytogenetic response with no Ph-positive metaphases and in major molecular response (MMR) with BCR-ABL1 negative. According to the National Comprehensive Cancer Network, all TKIs are highly effective in the newly diagnosed chronic phase of CML,^[9]^ and the vast majority of CML patients can achieve complete molecular remission with no BCR-ABL1 rearrangement using reverse transcription quantitative polymerase chain reaction (RT–qPCR) after TKI therapy.

## 2 Case report

### 2.1 Patient information

**Figure 1:**
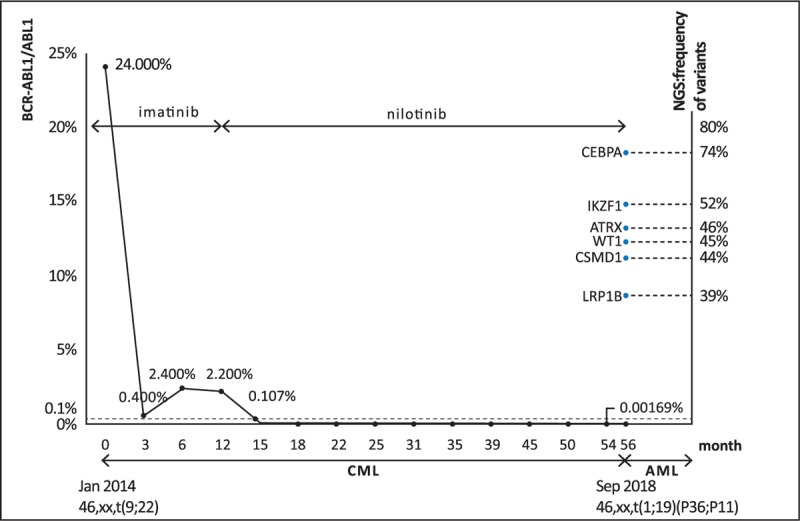
Diagnostic cytogenetics, next-generation sequencing of exome, BCR-ABL1 transcript levels, and therapy of TKIs from the diagnosis of CML to AML.

Here we describe a case of AML rising from chronic phase CML. The patient was a 56-year-old woman with a medical history of stable hypertension who was diagnosed as chronic phase CML after emerging hepatomegaly and splenomegaly. She was treated with TKI therapy (imatinib 400 mg/day) immediately after diagnosis and then monitored for durable MMR for nearly 3 years. The sole abnormal karyotype was t(9;22)(q34;q11)[12] at original diagnosis in January 2014. Bone marrow aspiration showed 4% blasts, and fluorescence in situ hybridization analysis of bone marrow cells revealed that the patient had 189 cells bearing BCR-ABL1 fusion (p210) from among 200 counted. At the same time, the ratio of BCR-ABL1 to ABL1 transcript numbers was 24.000%, standardized by an international scale (IS) using RT–qPCR. The patient first received TKI therapy (imatinib 400 mg/day). The patient's BCR-ABL1/ABL1 transcripts as monitored by RT–qPCR (IS) were 0.400%, 2.400%, and 2.200% at the 3-, 6-, and 12-month evaluations, respectively. After failing to achieve BCR-ABL1 transcripts <0.1% at 1 year after first-line therapy with imatinib, the therapy was changed to nilotinib 400 mg twice daily, which is associated with superior cytogenetic and molecular response rates compared with imatinib.^[10]^ After the BCR-ABL1 positive clone was not detectable for the first time in July 2015, the patient has achieved durable CCR and MMR. The monitoring of BCR-ABL1 using RT–qPCR (IS) has been performed continuously, after achieving BCR-ABL1 (IS) ≤1% (>0.1–1%), every 3 months for 2 years and every 3 to 6 months thereafter (Fig. 1).

**Figure 2:**
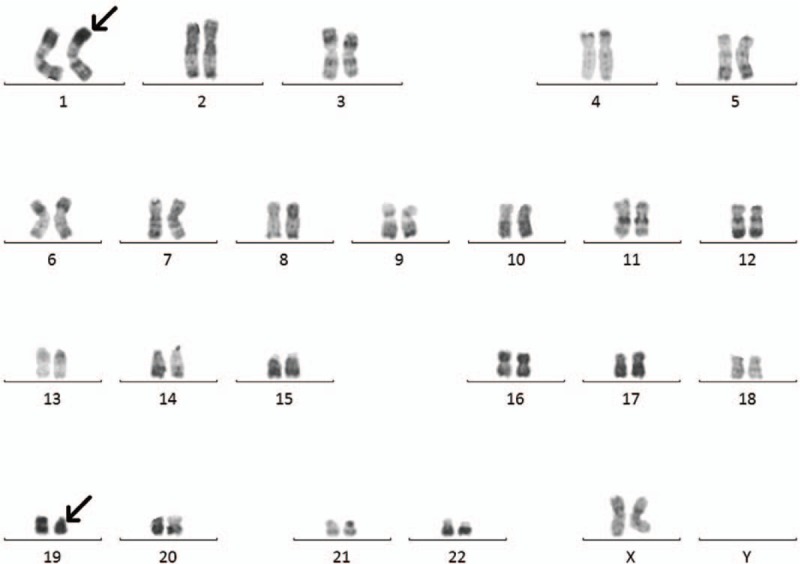
Karyogram shows t(1; 19)(p36; p11) [10] at 54 months after diagnosis of CML.

Consequently, in July 2018, a slight abnormality of 0.00169% was seen; however, no abnormality was seen in peripheral blood. Routine examination of peripheral blood has been normal, except once in September 2018, when pancytopenia was detected. Bone marrow aspiration was immediately performed, and the cytomorphology of bone marrow cells showed 45% myeloblast cells. The patient was thus newly diagnosed as having AML. At the same time, to our surprise, fluorescence in situ hybridization analysis of bone marrow cells showed a negative result for BCR-ABL1 fusion signal, and RT–qPCR using bone marrow cells showed no copies of BCR-ABL1 (p210) or mutational types of BCR-ABL1. Additionally, a sole karyotype abnormality other than Ph chromosome, a translocation between chromosome 1 and 19, t(1; 19)(p36; p11),^[10]^ was seen (Fig. 2).

Next-generation sequencing of peripheral cells to screen for 42 kinds of genetic mutations of leukemia showed CEBPA mutation at 67.63% and IKZF1 mutation at 50.20%. Since the novel mutation appeared after achieving MMR, we analyzed the patient's whole exome sequence. The results revealed 6 kinds of somatic variants—CEBPA, ATRX, WT1, CSMD1, IKZF1, and LRP1B mutation—accounting for 74.00%, 46.00%, 45.00%, 44.00%, 52.00%, and 39.00%, respectively. In other words, the patient newly developed AML with CCA/Ph– after achieving MMR of CML, and showed complete remission state of 4% myeloblast cells in bone marrow after receiving induction chemotherapy (idarubicin 10 mg/m^2^ d1–d3, cytarabine 100 mg/m^2^ d1–d7 continuous infusion). The patient, subsequently, has been treated with consolidation therapy of high dose cytarabine after she achieved complete response of AML. In addition, the woman has accepted nilotinib 400 mg twice daily for CML at the intermission of the chemotherapy before preparing to receive allogeneic hematopoietic stem cell transplantation from an HLA-matched donor.

## 3 Discussion

None of the above mutations detected in the phase of newly diagnosed AML have been found in any report of AML arising from CML treated with TKIs. Unfortunately, the gene mutation was not detected at the initial diagnosis or during therapeutic intervention for CML. Hence, we failed to represent the dynamic variation of the mutational gene from the beginning of the diagnosis of CML to that of AML. On the whole, the novel existence of gene mutations indicates various possibilities of the appearance of CCA/Ph–AML after achieving MMR in phase of CML.

Cases of CML progressing to CCA/Ph–MDS/AML after treatment with imatinib, dasatinib, or nilotinib have been reported.^[2,4,6,11]^ However, the pathogenesis of CCA/Ph–AML developing from CML has not been worked out completely yet. TKIs therapy for CML, to some extent, may indeterminately make influence on the appearance of CCA/Ph–MDS/AML. As is reported, the clonal cytogenetic changes of normal ABL protein caused by imatinib itself,^[12]^ possibly devoted to the mechanism of CCA/Ph–AML after CML with TKIs therapy. Besides, abnormal DNA damage repairs result in chromosomal changes due to cellular ABL kinase inhibited by TKIs.^[13]^ Additionally, as the case described here, the type of TKI drugs or therapeutic change is probably related to the occurrence of AML rising from CML with Ph chromosome negative. Early mutation(s) may already exist, according to 1 hypothesis, unknown, which then leading to AML after cooperating with BCR/ABL. On account of the prior attraction of BCR-ABL1 fusion, the latent clone mutation may play a leading role, developing into MDS/AML when the BCR-ABL1 fusion oncogene disappears or the Ph chromosome becomes negative. In addition, the study also suggested that abnormal bone marrow stroma may result in the acquisition of BCR–ABL1 and other genetic aberrations, leading to MDS or AML.^[6]^ Therefore, in addition to the molecular detection of BCL/ABL in CML, which used to be the main monitor for response to TKI therapy, cytogenetic analysis or next-generation sequencing of CML patients should be performed.

## Author contributions

**Formal analysis:** Jia Liu.

**Methodology:** Bin Yang.

**Resources:** Yun Ling.

**Software:** Wei Wu.

**Writing – original draft:** Huan Zhu.

**Writing – review & editing:** Yun Ling.
